# Pharmacological Potential of Cinnamic Acid and Derivatives: A Comprehensive Review

**DOI:** 10.3390/ph18081141

**Published:** 2025-07-31

**Authors:** Yu Tian, Xinya Jiang, Jiageng Guo, Hongyu Lu, Jinling Xie, Fan Zhang, Chun Yao, Erwei Hao

**Affiliations:** 1 Guangxi Key Laboratory of Efficacy Study on Chinese Materia Medica, Guangxi University of Chinese Medicine, Nanning 530000, China; 2 University Engineering Research Center of Reutilization of Traditional Chinese Medicine Resources, Nanning 530000, China; 3 Guangxi Key Laboratory of TCM Formulas Theory and Transformation for Damp Diseases, Guangxi University of Chinese Medicine, Nanning 530000, China; 4 Engineering Research Center of Innovative Drugs for Traditional Chinese Medicine and Zhuang & Yao Medicine, Ministry of Education, Guangxi University of Chinese Medicine, Nanning 530000, China

**Keywords:** Chinese medicine, cinnamic acid, cinnamic acid derivatives, pharmacological effects, mechanism of action

## Abstract

Cinnamic acid, an organic acid naturally occurring in plants of the Cinnamomum genus, has been highly valued for its medicinal properties in numerous ancient Chinese texts. This article reviews the chemical composition, pharmacological effects, and various applications of cinnamic acid and its derivatives reported in publications from 2016 to 2025, and anticipates their potential in medical and industrial fields. This review evaluates studies in major scientific databases, including Web of Science, PubMed, and ScienceDirect, to ensure a comprehensive analysis of the therapeutic potential of cinnamic acid. Through systematic integration of existing knowledge, it has been revealed that cinnamic acid has a wide range of pharmacological activities, including anti-tumor, antibacterial, anti-inflammatory, antidepressant and hypoglycemic effects. Additionally, it has been shown to be effective against a variety of pathogens such as *Staphylococcus aureus*, *Pseudomonas aeruginosa*, and foodborne Pseudomonas. Cinnamic acid acts by disrupting cell membranes, inhibiting ATPase activity, and preventing biofilm formation, thereby demonstrating its ability to act as a natural antimicrobial agent. Its anti-inflammatory properties are demonstrated by improving oxidative stress and reducing inflammatory cell infiltration. Furthermore, cinnamic acid enhances metabolic health by improving glucose uptake and insulin sensitivity, showing promising results in improving metabolic health in patients with diabetes and its complications. This systematic approach highlights the need for further investigation of the mechanisms and safety of cinnamic acid to substantiate its use as a basis for new drug development. Particularly in the context of increasing antibiotic resistance and the search for sustainable, effective medical treatments, the study of cinnamic acid is notably significant and innovative.

## 1. Introduction

Cinnamon is the dried bark of *Cinnamomum cassia* Presl (Camphoraceae), also known as fungus cinnamon or peony cinnamon, and is primarily produced in the Guangxi, Guangdong, and Hainan provinces of China [1]. Cinnamon has been utilized for centuries both as an herbal remedy and as a culinary spice [2]. In traditional medicine, cinnamon is an important medicinal herb for warming and tonifying yang, with its clinical applications primarily manifested in three main aspects: ① alleviating cold-induced pain in the lower back and knees, dysmenorrhea, and cold-induced abdominal pain by warming the meridians and promoting blood circulation; ② treating symptoms of yang deficiency (such as dizziness, ocular redness, and an imbalance of upper-body heat with lower-body cold) by guiding the fire back to its source; and ③ relieving cold-induced chest and abdominal pain, along with cold-type diarrhea, by dispersing cold and easing pain [2]. Modern pharmacological research has confirmed that the main active components of cinnamon, cinnamic acid and its derivatives, possess multiple pharmacological effects including anti-tumor, anti-inflammatory, antibacterial, anti-diabetic, and antioxidant properties [3,4,5,6,7]. These findings not only validate the rationality of its traditional applications but also provide scientific evidence for expanding its modern medical value.

Numerous studies have shown that cinnamic acid and its derivatives exhibit therapeutic effects against various types of cancer, including breast cancer [8], colon cancer [9], lung adenocarcinoma [10], prostate cancer [11], and chronic myelogenous leukemia [12]. In terms of their anti-inflammatory properties, cinnamic acid and its derivatives have been shown to inhibit the activation of Toll-like receptor 4 (TLR4) and nuclear factor kappa B (NF-κB) signaling pathways, leading to reduced production of pro-inflammatory cytokines such as TNF-α and IL-6. Research on the prevention and treatment of diabetes and its complications has highlighted cinnamic acid and its derivatives as having multiple mechanisms of action. These compounds have demonstrated the abilities to stimulate insulin secretion, improve the function of pancreatic beta cells, inhibit hepatic gluconeogenesis, and enhance glucose uptake. Consequently, they hold promise as a potential effective treatment for diabetes [13]. Cinnamic acid and its derivatives demonstrate varying levels of antibacterial activity against *Staphylococcus aureus*, *Pseudomonas aeruginosa*, foodborne Pseudomonas species, *Candida albicans*, and other fungal strains [14,15,16,17]. This report systematically integrates the latest research advancements in cinnamic acid and its derivatives published from 2016 to 2025. Building on a comprehensive summary of their traditional pharmacological effects, such as anti-inflammatory, antibacterial, and anti-tumor activities, it further extends to emerging application areas including antidepressant, hypoglycemic, neuroprotective, antioxidant, and anti-obesity effects. This in-depth, multidimensional analysis of molecular mechanisms lays a solid pharmacological foundation for the clinical translation of cinnamic acid and its derivatives.

## 2. Review Methodology

**Figure 1:**
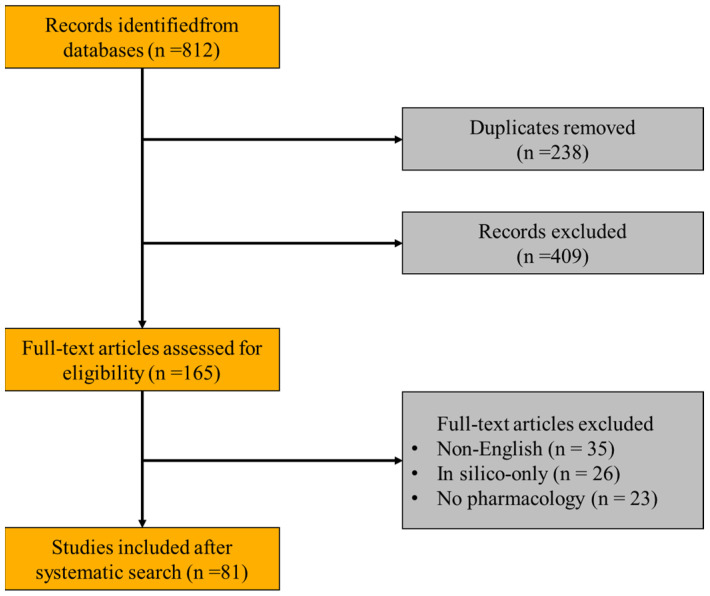
PRISMA flow diagram illustrating identification, screening, eligibility assessment, and inclusion of studies in this review.

To comprehensively review the pharmacological effects of cinnamic acid and its derivatives, a systematic search was conducted across major scientific databases including Medline, PubMed, ScienceDirect, and Scopus, guided by the PICOS principle. Specifically, PICOS criteria were defined as follows: P (population)—studies involving cinnamic acid, cinnamic acid derivatives, or related compounds; I (intervention)—administration of cinnamic acid and its derivatives in vitro, in vivo, or in clinical settings; C (comparison)—studies with or without controls (placebo or comparative treatment); O (outcome)—pharmacological effects, therapeutic efficacy, toxicity profiles, or mechanistic insights; and S (study design)—experimental studies, clinical trials, systematic reviews, and meta-analyses published in peer-reviewed journals. The detailed search strategy employed precise terms and syntax for database queries. For example, the following search terms and Boolean operators were used in PubMed: (“Cinnamic acid” [ MeSH Terms] OR “Cinnamic acid derivatives” [Title/Abstract] OR “caffeic acid phenethyl ester” [Title/Abstract] OR “CAPE” [Title/Abstract] OR “hydroxycinnamic acid” [Title/Abstract]) AND (“pharmacological effects” [Title/Abstract] OR “anti-inflammatory” [Title/Abstract] OR “anti-tumor” [Title/Abstract] OR “anti-bacterial” [Title/Abstract] OR “anti-diabetic” [Title/Abstract]) AND ((“1 January 2016” [Date–Publication]: “1 June 2025” [Date–Publication])). Similar structured queries were adapted for searches in Medline, ScienceDirect, and Scopus databases, adjusted slightly according to database-specific indexing and search features. Additionally, manual searches were performed by reviewing references cited within relevant articles to ensure comprehensive coverage of pertinent studies published between January 2016 and June 2025. The selection criteria for studies included in this review were clearly predefined. Inclusion criteria consisted of peer-reviewed original research articles, systematic reviews, meta-analyses, and clinical trial reports published in English. Studies reporting pharmacological actions, therapeutic efficacy, mechanistic details, or toxicity profiles of cinnamic acid and its derivatives, based on either in vitro or in vivo experiments or clinical studies, were considered eligible. Exclusion criteria explicitly comprised the following: studies published in languages other than English; studies based solely on in silico methods without experimental validation; articles primarily focused on chemical synthesis or analytical methods without reporting biological activity or pharmacological effects; and conference abstracts, editorial letters, opinion articles, and non-peer-reviewed publications. Initially, a total of 812 articles were identified through systematic database searching. After removing 238 duplicate records, 574 unique articles remained. Through title and abstract screening, 409 studies were further excluded due to irrelevance to pharmacological effects or failing to meet the inclusion criteria (such as being purely synthetic or analytical studies). The remaining 165 articles underwent full-text review, from which an additional 84 articles were excluded (35 articles due to language limitations, 26 due to being purely computational without biological verification, 23 due to lacking specific pharmacological or mechanistic information). Ultimately, 81 studies met all inclusion criteria through systematic search. Additionally, relevant references identified from citations, review articles, and expert recommendations were also included to provide comprehensive background information and discussion, resulting in a total of 127 references cited in this comprehensive review. The whole process flow is shown in Figure 1.

## 3. Chemical Composition

**Figure 2:**
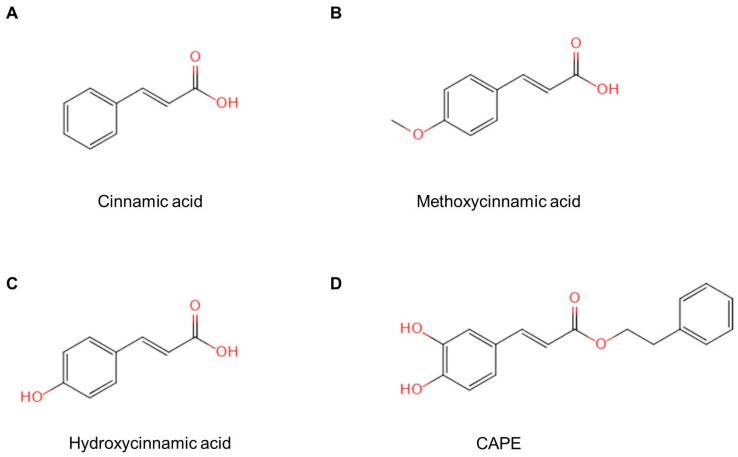
Chemical structures of cinnamic acid and its derivatives. (**A**) Cinnamic acid; (**B**) Methoxycinnamic acid; (**C**) Hydroxycinnamic acid; (**D**) CAPE.

Cinnamon has a broad spectrum of clinical applications. Cinnamic acid, a key aromatic carboxylic acid in cinnamon, has been widely studied both domestically and internationally. As extraction techniques for active ingredients advance, it has been discovered that cinnamic acid is found not only in cinnamon but also in various fruits, grains, vegetables, honey, and other natural sources. Cinnamic acid (Figure 2A) is an aromatic carboxylic acid characterized by a benzene ring conjugated with a propenoic acid side chain. It serves as the fundamental scaffold for numerous derivatives, including methoxycinnamic acid, hydroxycinnamic acid, and caffeic acid phenethyl ester (CAPE), each bearing specific substituents on the aromatic ring or modifications on the aliphatic chain (Figure 2B–D).

In addition to the parent compound, cinnamic acid, several notable derivatives have been widely investigated due to their potential pharmacological benefits. For instance, trans-cinnamic acid (t-CA) differs from cis-cinnamic acid (c-CA) by the configuration around the double bond, whereas caffeic acid phenethyl ester (CAPE) contains additional hydroxyl groups on the aromatic ring, enhancing its antioxidant properties. 3,4,5-trihydroxycinnamic acid decyl ester introduces multiple hydroxyl substituents, which have been associated with increased pro-apoptotic activity in certain cancer cell lines.

To systematically compare these derivatives, Table 1 summarizes their key physicochemical properties, including molecular weight, pKa, and solubility profiles. Notably, substituents such as methoxy (–OCH_3_) or hydroxy (–OH) groups can increase the polarity and hydrogen-bonding capacity, potentially influencing both bioavailability and interactions with molecular targets.

To visualize the structure–activity relationships beyond the physicochemical comparison provided in Table 1, Table 2 lists the principal cinnamic acid derivatives, their characteristic substituents, validated molecular targets, and translational highlights.

These data emphasize how minor structural changes—such as hydroxylation, nitro-substitution or esterification—pivotally redirect target engagement, providing a guide for future optimization of cinnamic acid scaffolds.

Previous studies have reported that introducing phenolic hydroxyl groups enhances antioxidant and anti-inflammatory activities by increasing the hydrogen-donating ability [7]. Furthermore, certain esterification modifications (e.g., decyl ester) may improve lipophilicity, thereby facilitating cellular uptake [3]. These variations in chemical structure underlie the diverse bioactivities covered in subsequent sections.

Given its promising research potential, cinnamic acid can be synthesized via deamination of phenylalanine to meet clinical demands [18]. As summarized in Table 1, cinnamic acid serves as the foundational scaffold for various derivatives, each exhibiting unique physicochemical characteristics that can influence their therapeutic applications. Beyond its occurrence in cinnamon bark, cinnamic acid is used in multiple industries, including medicine, food, and cosmetics. It also functions as a fragrance ingredient in detergents and personal care products [19].

In conclusion, the structural diversity of cinnamic acid derivatives underpins their varied bioactivities. The following sections explore how these structural modifications correlate with anti-inflammatory, antibacterial, and anticancer mechanisms, illustrating the potential for targeted therapeutic application.

## 4. Anti-Inflammatory Effect

**Figure 3:**
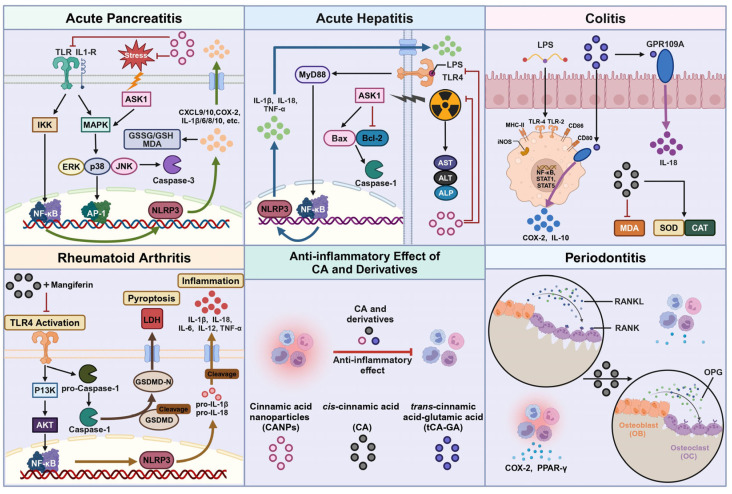
Anti-inflammatory effect of CA and its derivatives. (Created with BioRender.com).

Inflammation is the body’s immune response to pathogens, toxic substances, and environmental stressors [20]. If left unresolved, inflammation can lead to chronic pathological conditions that perpetuate immune cell activation and tissue damage [21]. Therefore, the search for new anti-inflammatory therapeutic options is crucial. Cinnamic acid, a naturally occurring compound, has attracted significant research interest for its anti-inflammatory properties. Exploring cinnamic acid and its derivatives as potential anti-inflammatory agents is thus of great importance (Figure 3).

### 4.1. Anti-Acute Pancreatitis

Acute pancreatitis is an inflammation triggered by the abnormal activation of pancreatic digestive enzymes [22].

Due to its complex etiology, numerous complications, and high mortality rate, acute pancreatitis leads to moderate to severe disease in roughly 20% of patients. These symptoms typically manifest as acute abdominal pain, bloating, nausea, vomiting, and fever [23]. Research has shown that radiation injury can trigger pancreatitis. For example, abdominal radiotherapy can cause acinar cell necrosis, mild ductal cell damage, duct atrophy, vasculopathy, and diffuse delayed fibrosis in the pancreas [24]. Therefore, exposure to radiation is now recognized as an important risk factor for pancreatitis.

Omayma AR Abozaid’s [25] study discovered that cinnamic acid nanoparticles (CA-NPs) have a therapeutic impact on rats suffering from acute pancreatitis caused by L-arginine and gamma rays. The researchers observed that pancreatitis-induced rats exhibited elevated malondialdehyde (MDA) levels and an increased pancreatic ratio of oxidized to reduced glutathione (GSSG/GSH), indicating heightened oxidative stress. Oral administration of CA-NPs effectively counteracted this oxidative stress, reducing MDA and normalizing GSSG/GSH levels. However, oral administration of CA-NPs effectively reduced oxidative stress. Additionally, L-arginine significantly activated ERK1/2, JNK, and p38 in MAPK signaling pathways, while CA-NPs administration inhibited these pathways, demonstrating anti-inflammatory properties. In terms of the inflammatory response in acute pancreatitis, NF-κB and NLRP3 gene expression levels were significantly elevated, but CA-NPs administration led to a significant decrease in the transcription levels of these genes. The NF-κB signaling pathway plays a central role in inflammation and tumorigenesis by regulating cytokines, chemokines, and genes involved in cell survival and proliferation. Cinnamic acid derivatives such as CA-NPs significantly inhibit NF-κB signaling by reducing phosphorylation of IκB, thus decreasing pro-inflammatory gene transcription (such as TNF-α, IL-6). This NF-κB inhibitory activity is crucial for both their anti-inflammatory and anti-tumor effects. The study identified caspase activation as a key apoptotic mechanism in pancreatitis, evidenced by increased levels of the pro-apoptotic protein caspase-3 and upregulated ASK1 expression during acute pancreatitis. Treatment with CA-NPs effectively reversed L-arginine’s inflammatory activation, suggesting a potential role for CA-NPs in protecting cells from apoptosis.

In summary, CA-NPs alleviate the severity of acute pancreatitis through NLRP3 gene downregulation, and mitigate oxidative stress and inflammatory responses by inhibiting the NF-κB and ASK1/MAPK signaling pathways. Based on this mechanism, CA-NPs demonstrate potential as a therapeutic agent for acute pancreatitis. However, inherent physiological and metabolic differences between animal models and humans necessitate further validation of their translational potential and safety in more human-relevant models and clinical trials.

### 4.2. Anti-Acute Hepatitis

Hepatitis is a global public health concern caused by autoimmune diseases, alcohol consumption, metabolic disorders, viral infections, and fatty liver disease [26]. Acute hepatitis is characterized by severe inflammation, which can result in liver cell death and ultimately lead to liver failure, posing a serious threat to human health and survival [27].

Ehab A. Ibrahim et al. [28] conducted a study on the regulatory effect of cinnamic acid nanoparticles (CA-NPs) on d-galactosamine (D-Gal) and radiation-induced acute hepatitis in rats. The study indicated that administering d-galactosamine/radiation (AH group) via intraperitoneal injection resulted in a significant increase in serum ALT, AST, and ALP activities. However, following oral administration of CA-NPs, there was a notable reduction in the activities of these transaminases and ALP when compared to the AH group(*p* < 0.05). Experimental research found a significant increase in TNF-α, IL-1β, and IL-18 levels in liver tissue of the AH group, as well as elevated gene expressions of IL-1βmRNA and IL-18 mRNA. Oral administration of CA-NPs led to a notable decrease in inflammatory cytokine levels, IL-1β and IL-18 gene expressions, and mitigated the induction of TLR4 and MyD88 levels in the AH group. Analysis showed that CA-NPs effectively reduced NLRP3 and NF-κB levels in liver tissue. As noted earlier (see Section 4.2), cinnamic acid derivatives strongly inhibit NF-κB signaling, thereby suppressing downstream inflammation-related pathways and producing broad anti-inflammatory effects. Furthermore, CA-NPs decreased Caspase-1, BAX, and ASK1 gene expressions while increasing Bcl-2 levels, thus reducing cell apoptosis.

Experimental observations have demonstrated that acute hepatitis induced by D-galactosamine/radiation leads to hepatic cord tissue disorder and multifocal hepatic coagulative necrosis throughout the liver lobule, with Kupffer cells, lymphocytes, and macrophages proliferating up to grade IV levels. Oral administration of CA-NPs before D-Gal/irradiation induction significantly improved the liver tissue, normalizing the structure of lobules and reducing Kupffer cell proliferation to level 0.

In summary, CA-NPs exhibit significant hepatoprotective effects in the D-Gal and radiation-induced acute hepatitis rat model by inhibiting the MyD88/TLR4 pathway, providing experimental evidence for their clinical application in treating acute hepatitis. However, due to inherent physiological and metabolic differences between rodent models and humans in terms of etiology, disease progression, and drug response, direct translation of these findings to clinical practice requires caution. Future systematic clinical trials are necessary to further validate the efficacy and safety of CA-NPs.

### 4.3. Anti-Colitis

Inflammatory bowel disease (IBD) is a chronic and relapsing inflammatory disease of the gastrointestinal tract, including ulcerative colitis (UC) and Crohn’s disease [29]. At present, there are no medications available to cure IBD, and existing anti-IBD drugs, such as aminosalicylates, glucocorticoids, and immunosuppressants, are only capable of alleviating symptoms [30]. To solve the problems of low efficacy and serious side effects caused by long-term use [31], there have been recent advancements in the treatment of IBD with the introduction of anti-tumor necrosis factor, anti-integrin, and anti-IL-12 and IL-23 biological agents [32]. Biological agents, although effective, have drawbacks such as common side effects, high drug costs, non-parenteral administration methods, which lead to poor patient compliance and potential development of increased tolerance in patients [33]. To enhance the efficacy and reduce the toxicity of conventional drugs, it is essential to thoroughly research and develop small molecule anti-inflammatory bowel disease (IBD) drugs. During the investigation of potential natural products for colitis treatment, cinnamic acid (CA) was identified as a promising therapeutic agent.

Maysam A Hussein et al. [4] conducted research to investigate the protective effect of cinnamic acid on ulcerative colitis induced by dextran sodium sulfate in mice. MDA is a critical indicator used to quantify oxidative damage levels in the body [34]. In comparison to the control group, levels of MDA showed a significant increase, whereas total levels of superoxide dismutase (SOD) and catalase (CAT) exhibited a notable decrease, resulting in the development of ulcerative colitis. The experiment used cinnamic acid suspension for seven consecutive days. The research findings indicate that cinnamic acid has the potential to reduce ulcerative colitis (UC) induced by dextran sodium sulfate (DSS) by reducing MDA levels and increasing total SOD and CAT levels. Additionally, the histopathological examination revealed a notable decrease in inflammatory symptoms in the cinnamic acid-treated group compared to the DSS model group.

Changyu Kang et al. [35] formulated a colon-targeted drug, tCA-GA, by combining trans-cinnamic acid (t-CA) and glutamic acid to analyze the anti-inflammatory effect on dinitrobenzene sulfonic acid (DNBS)-induced colitis in rats. GPR109A has been identified as a crucial target for the treatment of colitis. Its activation inhibits the NF-κB pathway. As noted earlier (see Section 4.2), cinnamic acid derivatives strongly inhibit NF-κB signaling, thereby suppressing downstream inflammation-related pathways and producing broad anti-inflammatory effects, reducing the release of pro-inflammatory factors while simultaneously promoting T cell function and IL-10 secretion to modulate immune responses. Additionally, it enhances intestinal barrier function to alleviate inflammatory damage. Research has shown that tCA-GA is capable of activating GPR109A. The experiment demonstrated that oral administration of tCA-GA effectively suppressed colon inflammation induced by DNBS by reducing MPO activity and levels of inflammatory mediators CINC-3, iNOS, and COX-2 in the distal colon. However, the inhibitory effect of tCA-GA on colon inflammation was weakened by the administration of mepenzolate (MPZ). To further explore the involvement of GPR109A activation in the anti-colitis activity of tCA-GA, IL-10 levels in the inflamed colon were measured. The results showed that oral tCA-GA administration led to an increase in IL-10 levels in colitis, whereas co-administration of MPZ significantly decreased IL-10 levels in the inflamed colon of rats. These findings suggest that tCA-GA exerts its anti-colitis effect by activating GPR109A.

Collectively, cinnamic acid and its derivatives are promising drugs for the treatment of colitis, and these results offer novel insights on the functions of cinnamic acid and its derivatives in colitis treatment.

### 4.4. Anti-Rheumatoid Arthritis

Rheumatoid arthritis (RA) is a refractory and highly prevalent autoimmune disease [36]. The main pathological features of the disease include chronic persistent synovitis, synovial cell hyperplasia (FLS), inflammatory cell infiltration (including monocytes, polyclonal B cells, and T cells), erosion, and bone tissue destruction [37]. These factors contribute to structural damage in the joints, deformities, and eventual loss of function [38]. At present, the primary therapies for RA consist of surgical intervention and medication management [39], including disease-induced anti-rheumarthritis drugs (DMARDs), nonsteroidal anti-inflammatory drugs (NSAIDs), glucocorticoids, and biological response modifiers. While these medications have the potential to diminish synovitis and systemic inflammation, they might still be less efficacious for certain patients [40]. Hence, gaining a comprehensive understanding of RA’s pathogenesis, identifying novel therapeutic targets, and developing innovative therapeutic drugs is crucial.

To investigate the pharmacological impact of the joint administration of cinnamic acid (CA) and mangiferin (MG) on rheumatoid arthritis, a study was carried out by Weijie Li [41] using an adjuvant-induced arthritis modified (AIA-M) rat model of RA. The combined therapy notably reduced ankle joint swelling and overall disease severity in these rats. Including reducing the incidence of arthritis, limb diameter, and arthritis score, while improving the pain threshold (mechanical, Acetone, and heat-induced hyperalgesia), its efficacy is similar to that of the positive drug methotrexate (MTX). To further investigate the impact of rheumatoid arthritis on joint destruction and synovial inflammation, micro-CT scans were conducted on the ankle and knee joints of various groups of AIA-M rats. The findings revealed a significant decrease in BMD, TMD, BV/TV ratio, Tb, and Th in the model group, along with roughening of the bone surface, severe bone erosion, and increased Sp (*p* < 0.05). Conversely, the combined treatment group demonstrated a restoration of bone surface smoothness, a significant increase in BMD, TMD, BV/TV ratio, and Tb, and a decrease in Sp (*p* < 0.05). These results suggest that the combined treatment effectively reverses bone erosion and exhibits pharmacological effects similar to the positive drug MTX. The study utilized hematoxylin and eosin (H&E), safranin O-fast green, and Masson’s trichrome staining to evaluate knee joint disease severity in AIA-M rats. Results show that the combined therapy effectively reduces inflammatory cell infiltration, prevents cartilage and bone damage, and decreases synovial hyperplasia. Additionally, the treatment significantly mitigated arthritis-induced bone damage in AIA-M rats, repaired bone erosion, and reduced cartilage lesions and synovial inflammation. Evaluation of thymus and spleen pathology in all rat groups revealed that AIA-M rats exhibited decreased white pulp in the spleen, diminished density of lymphatic sheath cells, reduced hyperplasia of lymphoid follicles, thinned the thymic cortex, reduced thymic lobules, diminished indistinct boundaries, and increased vacuoles in epithelial reticular cell cytoplasm. Treatment with the combination significantly ameliorated these pathological alterations and preserved the structural integrity of the spleen and thymus.

The experiment was based on network pharmacology results and suggested that the combination treatment may work by regulating the TLR4/PI3K/AKT/NFkB/NLRP3 signaling pathway. Analysis of the results showed that in rats with AIA-M, the combined treatment effectively reversed the increased TLR4 expression levels in serum and knee joints. Additionally, the combined treatment suppressed the activation of the TLR4/PI3K/AKT/NFkB pathway and reduced levels of inflammatory cytokines TNF-a, IL-6, and IL-12.

In summary, the combination of CA and MG effectively inhibits the activation of proteins involved in the TLR4/PI3K/AKT/NF-κB signaling pathway, decreases caspase-1 expression, and reduces IL-1β release, resulting in the alleviation of arthritis symptoms and bone erosion. This finding offers a fresh pathway for further detailed investigation into the mechanism of cinnamic acid in treating rheumatoid arthritis, consequently advancing the modernization of traditional Chinese medicine and the development of new drugs.

### 4.5. Anti-Periodontitis

Periodontitis is a chronic and destructive disease affecting the periodontal tissues [42]. Its development is associated with an imbalance in oral microbiota and the host’s inflammatory response [43]. The level of microbial plaque accumulation is directly linked to the extent of inflammation, which in turn dictates the severity of the disease [44]. By addressing the inflammatory state, it is possible to halt the progression of periodontal destruction and enhance periodontal health. Therefore, the development of novel drugs for treating periodontitis holds significant importance.

Cinnamic acid, as an unsaturated carboxylic acid [45], plays a crucial role in the prevention of inflammation and periodontal destruction. In a study conducted by Ozkan Karatas [46], higher expression levels of PPAR-γ, COX-2, and RANKL were observed in Wistar rats with ligation-induced periodontitis, along with an increased inflammatory response and osteoclast count. Conversely, the expression of OPG was lower and osteoblast counts were reduced. Following treatment with cinnamic acid, a significant decrease in the expression of RANKL, inflammation, and the number of osteoclasts was noted, leading to reduced bone loss. Moreover, there was an increase in the expression of OPG and the number of osteoblasts, accompanied by a notable decrease in the levels of PPAR-γ and COX-2.

In summary, oral administration of cinnamic acid was shown to have a significant preventive impact on periodontitis and also helped in suppressing the host’s inflammatory response. These results suggest that cinnamic acid could potentially be an effective adjunctive treatment or preventive medication for periodontitis.

## 5. Antibacterial Effect

China has an abundance of cinnamon resources. Cinnamic acid, a constituent of cinnamon, has demonstrated the ability to inhibit the growth of various bacteria and fungi, making it a valuable natural antibacterial and antiseptic agent [47,48]. Therefore, understanding the mechanisms by which cinnamic acid and its derivatives combat pathogenic microbes is crucial for protecting human health.

### 5.1. Anti-Staphylococcus aureus

*Staphylococcus aureus* is one of the common opportunistic bacterial pathogens colonizing humans and causes significant morbidity and mortality worldwide [49]. Some strains can produce staphylococcal enterotoxins [50]. In China, a quarter of bacterial food poisoning cases are caused by *Staphylococcus aureus*. Recent research has shown an increasing antibiotic resistance in *Staphylococcus aureus* [51], leading to the emergence of multidrug-resistant strains (MRSA). Consequently, the search for new antibacterial agents has become a top priority. Cinnamic acid, a natural plant extract, is known for its significant antibacterial activity, particularly its inhibitory effect on *Staphylococcus aureus*.

Research indicates that the assembly of berberine (BBR) and flavonoid glycosides into nanoparticles (NPs) enhances their antibacterial efficacy against *Staphylococcus aureus* [52]. Huang Xuemei et al. [53] synthesized cinnamic acid (CA) and berberine (BBR) into organic nanostructures (CA-BBR NPs) to evaluate their efficacy against methicillin-resistant *Staphylococcus aureus* (MRSA). The minimum inhibitory concentration (MIC) of CA-BBR NPs was reported to be 0.075 μmol/mL, which is significantly lower than that of BBR alone (0.1 μmol/mL). This enhanced antibacterial efficacy highlights the synergistic action of CA and BBR in inhibiting MRSA growth and biofilm formation. As expected, the MRSA strain in this study was resistant to multiple first-line antibiotics (e.g., ofloxacin, amoxicillin, and tetracycline). In contrast, the CA-BBR NPs showed strong, dose-dependent inhibition of MRSA growth, outperforming those standard antibiotics. Notably, at 0.075 μmol/mL, CA-BBR NPs achieved an antibacterial effect greater than that of BBR alone, whereas the conventional antibiotics each had an antibacterial rate below 30% at that concentration. Furthermore, both the minimum inhibitory concentration and minimum bactericidal concentration of CA-BBR NPs were 0.075 μmol/mL, whereas those of BBR were 0.1 μmol/mL and 0.15 μmol/mL, respectively. The assessment of bacterial growth kinetics indicated a significant impact of CA-BBR NPs on the growth rate and activity level of bacteria, indicating that they play a key role in inhibiting bacterial activity.

The biological safety of CA-BBR NPs was evaluated through in vitro hemolysis, cytotoxicity, and in vivo zebrafish model studies. Results showed that CA-BBR NPs did not induce significant cytotoxicity on MDCK cells within the concentration range of 1.5625 to 50 μM after 24 and 48 h, with cell viability exceeding 82% at 50 μM. Hemolysis tests demonstrated no observable damage to rat red blood cells following administration of CA-BBR NPs, indicating their biocompatibility for potential blood-contact applications. Zebrafish model tests indicated that exposing healthy 1-day-old zebrafish to a solution of CA-BBR NPs for 72 h did not lead to mortality, spinal cord curvature, or morphological changes, suggesting minimal impact on the growth of zebrafish larvae. Overall, these findings collectively support the safe and biocompatible nature of CA-BBR NPs.

Biofilms are formed by surface-associated bacterial communities enveloped in an extracellular polysaccharide matrix, providing resistance to environmental and chemical damage. The formation of biofilms triggers extensive adaptive responses in bacteria. Recent studies have highlighted the increasing resistance of bacterial biofilms to traditional antibiotic therapies [54]. Consequently, experiments were carried out to assess the efficacy of CA-BBR NPs in eliminating MRSA biofilms. The findings demonstrated that at 0.1 μmol/mL, CA-BBR NPs achieved a 64.2 ± 3.0% MRSA biofilm removal rate, which was significantly higher than the 32.9% removal observed with BBR alone. SEM analysis confirmed that, at this concentration, CA-BBR NPs surpassed the BBR group in biofilm removal. Additionally, staining with the LIVE/DEAD^®^ BacLightTM Bacterial Viability Kit revealed notably higher red fluorescence intensity in the CA-BBR NPs group compared to the control and BBR groups, confirming the notable biofilm removal ability of CA-BBR NPs.

A study employed FESEM and TEM to observe the microscopic morphology of bacteria, investigate the interaction between CA-BBR NPs and MRSA, and explore the antibacterial mechanism of CA-BBR NPs on MRSA. FESEM results revealed that CA-BBR NPs could selectively encapsulate bacteria, a phenomenon not observed in the BBR group. Zeta-potential measurements were conducted for NPs in both aqueous solution and Luria–Bertani (LB) medium, showing a zeta potential of −32.1 mV in the aqueous solution and 2.96 mV in the LB medium. This indicated the affinity of CA-BBR NPs towards the negatively charged bacterial surface, leading to inhibition of nutrient intake, biofilm formation, and physiological metabolism, ultimately hindering bacterial growth. Examination of untreated bacteria showed spherical shapes with smooth and intact cell walls, while 12 h after the administration of CA-BBR NPs, bacterial cell walls displayed damage and wrinkles, accompanied by significant leakage of intracellular materials.

In summary, cinnamic acid exhibits potent activity against *Staphylococcus aureus*, including drug-resistant strains, through mechanisms such as bacterial cell membrane damage and biofilm inhibition. As a result, cinnamic acid shows promise as a therapeutic agent for combating *S. aureus* infections.

### 5.2. Anti-Pseudomonas aeruginosa

*Pseudomonas aeruginosa* (PA) is an aerobic Bacillus characterized by motility and a unipolar flagellum [55], It is also a Gram-negative opportunistic pathogen [56], responsible for causing a variety of systemic diseases, particularly affecting the digestive and respiratory systems. The overuse of antibiotics has led to the emergence of multi-drug or even pan-drug resistant *Pseudomonas aeruginosa*, presenting significant challenges for clinical treatment [57]. In burn patients, wound infections represent a major risk [58]. Burn wounds often become infected with antibiotic-resistant strains of *Pseudomonas aeruginosa*, exacerbating the wound condition and complicating treatment [59]. The excessive use of antibiotics has resulted in the development of biofilms by *Pseudomonas aeruginosa*, further complicating effective treatment [60]. Carbapenem antimicrobials are commonly reserved as a final resort for treating *Pseudomonas aeruginosa* (PA) infections. Nevertheless, there is a growing trend in resistance to these drugs in PA strains. With the diminishing efficacy of traditional antibacterial treatments, there is an urgent need for the advancement of new therapeutic strategies. To address this issue, scientists are investigating the utilization of natural products in wound care to alleviate the negative impacts of antibacterial medications and provide novel treatment alternatives.

In a study by Rajkumari et al. [61], the inhibitory effects of cinnamic acid on *Pseudomonas aeruginosa* were explored. The research revealed that cinnamic acid was effective in reducing the production of exovirulence factors in *Pseudomonas aeruginosa* at a sub-MIC concentration of 250 μg/mL. Specifically, it decreased pyocyanin production by 80.94%, total protease activity by 71.38%, elastase activity by 21.81%, and chitinase activity by 54.86%. The impact of cinnamic acid on biofilm formation of *Pseudomonas aeruginosa* PAO1 was assessed using the microtiter plate (MTP) method. The results showed a 50.13% decrease in biofilm formation under sub-MIC level cinnamic acid treatment. Moreover, rhamnolipid production decreased to 16.51%, and alginate production was significantly reduced by 49.86%. Additional research revealed that following the administration of CA, a 42.19% reduction in accumulated shared proteoglycans (CSH) on cell surfaces and a 25.14% decrease in exogenous DNA (eDNA) were observed. The TTC reduction assay showed that CA, at sub-MIC concentrations, decreased viable cell numbers in the biofilm matrix by 7.13% and inhibited the production of polysaccharide substances (EPS) in *Pseudomonas aeruginosa*. Confocal laser scanning microscopy (CLSM) analysis revealed that *P. aeruginosa* formed dense and compact structures across its surface, but post-CA treatment, there was a significant decrease in cell surface coverage. Z-slice analysis using CLSM demonstrated that the untreated *Pseudomonas aeruginosa* biofilm had an average thickness of 30 μM, whereas CA treatment reduced the biofilm thickness to 18 μM.

Ariana S.C. Gonçalves et al. [62] found that cinnamic acid derivatives, ferulic acid and caprylic acid, significantly inhibited the quorum sensing (QS) of *Pseudomonas aeruginosa*, reduced its biofilm thickness, and suppressed the secretion of virulence factors and bacterial motility. These results indicate that ferulic acid and caprylic acid effectively inhibited bacterial biofilm formation and virulence expression by blocking the QS pathway.

In summary, cinnamic acid and its derivatives demonstrate an inhibitory impact on *Pseudomonas aeruginosa* by affecting the production of virulence factors associated with disrupting the bacterial quorum sensing system and biofilm formation. These results provide a strong basis for future investigations into the potential use of cinnamic acid as an alternative to conventional antibacterial agents.

### 5.3. Anti-Foodborne Pseudomonas

Psychrophilic foodborne Pseudomonas is a Gram-negative, facultative anaerobic bacterium that thrives between 2 °C and 35 °C [63], leading to food spoilage [64] and significant economic repercussions in the food industry [65]. These bacteria contribute to the decline in food quality through the activity of extracellular proteases and lipases in products like milk, meat, and seafood within the cold chain [66]. Consequently, it is crucial to implement effective preventive strategies to mitigate economic losses in cold-chain food.

Due to the limitations of traditional heat treatment under low temperature conditions, plant polyphenols, as natural products, have become the focus of extensive research and play an important role in food [67]. Yuxiang Zhang et al. [16] found that cinnamic acid (CA) exhibits good antibacterial activity, with an inhibitory concentration of 0.25 mg/mL. The presence of CA significantly slows or even inhibits the growth rate of the strain. The antibacterial mechanism of CA was investigated through an experiment assessing cell membrane potential, intracellular pH value, and intracellular ATPase activity, and observing changes in the cell membrane. Results showed that without drug administration, the cell membrane’s resting potential remained stable. However, after administering CA, there was clear depolarization of the cell membrane and a significant increase in fluorescence intensity. The intracellular pH value is an indicator of cellular homeostasis. When CA concentration reached 4 MIC, the cellular equilibrium was disrupted, causing the pH value to drop to 4.71. ATPase, a key enzyme on cell and organelle membranes, is crucial for cellular material transport, energy conversion, and information transmission, maintaining normal physiological cell metabolism. As the CA concentration increased, there was a notable decrease in ATPase activity, disrupting cell membrane homeostasis and interfering with energy metabolism processes. Examination of the bacterial cell membrane post-treatment with various concentrations of CA revealed structural alterations in the membrane, contributing to cell damage and an increase in bacterial mortality rate that directly correlated with the level of CA concentration. Hence, CA has the capacity to induce cell membrane impairment, consequently leading to bacterial demise. Consequently, CA is recognized as a potent natural antibacterial agent.

In summary, cinnamic acid demonstrates notable antibacterial activity against food-borne Pseudomonas. Its primary antibacterial mechanism involves compromising the integrity of the cell structure, leading to disruption in the cell membrane’s homeostasis. Furthermore, it disrupts the intracellular energy metabolism of the bacteria. These findings suggest that cinnamic acid holds promise as a potential drug candidate against foodborne Pseudomonas species.

### 5.4. Antifungal

Fungal infections pose a significant challenge to global public health, particularly with the emergence of drug-resistant strains such as Candida and *Aspergillus fumigatus*, which have led to a reduced efficacy of traditional antifungal drugs [68,69]. Natural products and their derivatives are gradually becoming an important direction in the development of antifungal drugs due to their unique structural diversity and lower toxicity characteristics. Among these, cinnamic acid derivatives have garnered considerable attention in the field of antifungal drug development due to their excellent inhibitory effects against drug-resistant strains.

Tamires C. Lima et al. [70] investigated the antimicrobial effects of cinnamic acid derivatives against three strains of *Candida albicans* (ATCC-76645, LM-106, and LM-23). By determining the minimum inhibitory concentration (MIC) of the cinnamic acid derivatives, the study found that methyl caffeate and methyl 2-nitro cinnamate exhibited the most potent antimicrobial effects against the three strains of *Candida albicans*, both exhibiting an MIC of 128 μg/mL, which was lower than the MICs of other cinnamic acid derivatives. This finding indicates that methyl caffeate and methyl 2-nitro cinnamate possess certain antifungal activity against *Candida albicans*.

Jong H. Kim et al. [71] investigated the potential of cinnamic acid derivatives as intervention catalysts to overcome antifungal tolerance. The study evaluated the antifungal effects of 33 cinnamic acid derivatives using in vitro yeast dilution bioassays. The results indicated that 4-chloro-α-methylcinnamic acid and 4-methylcinnamic acid demonstrated the strongest antifungal activity against *Saccharomyces cerevisiae* cell wall integrity mutants. Furthermore, 4-chloro-α-methylcinnamic acid was able to overcome the tolerance of *Aspergillus fumigatus* antioxidant mitogen-activated protein kinase (MAPK) mutants to fipronil, while simultaneously inhibiting the growth of these MAPK mutants. Additionally, the combination of 4-chloro-α-methylcinnamic acid or 4-methylcinnamic acid with the antifungal agents caspofungin or octyl gallate reduced the minimum inhibitory concentration of these antifungal drugs, demonstrating their chemosensitizing capabilities. In summary, both 4-chloro-α-methylcinnamic acid and 4-methylcinnamic acid exhibit antifungal effects by enhancing the efficacy of antifungal drugs, thus holding potential for development as novel antifungal intervention catalysts.

In summary, cinnamic acid derivatives exhibit certain antifungal effects against drug-resistant strains, providing a theoretical basis for the development of new antifungal drugs in the future.

## 6. Anti-Tumor Effect

**Figure 4:**
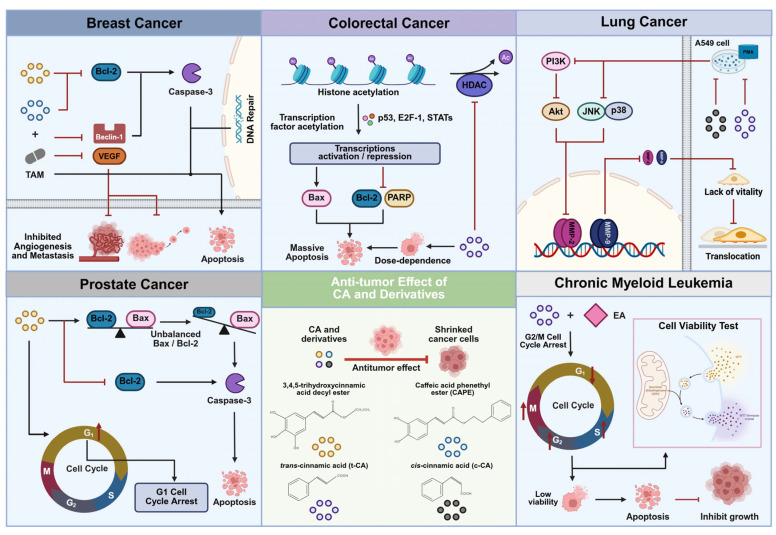
Anti-tumor effect of CA and derivatives. (Created with BioRender.com).

Cancer, a primary cause of mortality globally, arises when tumor tissue aggressively infiltrates adjacent healthy tissue and establishes secondary tumors (metastasis) in different organs [72]. The incidence and mortality rates of malignant tumors are increasing, presenting a substantial threat to human health and becoming a crucial public health concern requiring worldwide focus. Presently, the primary method in anti-tumor therapy revolves around the utilization of small molecule drugs. These treatments have achieved remarkable success and have played a vital role in extending the lives of individuals with cancer [73]. However, in clinical practice, anti-tumor drugs commonly encounter resistance issues [74], making it imperative to explore and create alternative drugs with higher efficacy and fewer side effects to effectively halt the progression of cancer (Figure 4).

### 6.1. Breast Cancer

Breast cancer is the most common malignant tumor and a leading cause of cancer-related mortality among women [75]. The latest data shows that newly diagnosed cases of breast cancer worldwide have reached 2.26 million, surpassing lung cancer to become the most common type of cancer [76]. The prognosis of breast cancer is more favorable in the early stage; however, challenges persist in the middle and late stages [77]. Over recent years, there has been a rising incidence of breast cancer in China, particularly among younger individuals [76]. Although multidisciplinary treatment has shown promise in enhancing patient survival rates, it also brings about substantial psychological and physical strain on patients. Studies have indicated potential inhibitory properties of cinnamic acid derivatives on breast cancer progression, yet further research is required to comprehensively grasp the underlying mechanisms and targets.

Masahiko Imai et al. [11] conducted a study using the MTT method to evaluate the effects of 3,4,5-trihydroxycinnamate decyl ester, a cinnamic acid derivative, on the growth of MCF-7 breast cancer cells. The results indicated that the compound exhibited an IC_50_ value of ~3.2 µM, effectively inhibiting MCF-7 cell viability. At a concentration of 4 µM, the drug inhibited MCF-7 cell growth by approximately 98%, demonstrating a significant inhibitory effect on breast cancer cell proliferation. Additionally, the study investigated the mechanism of this inhibition by utilizing flow cytometry to analyze the cell cycle. The findings revealed a dose-dependent increase in the number of cells in the G1 phase of MCF-7 cells after treatment. Furthermore, the drug was found to enhance caspase-3 activity in MCF-7 cells while decreasing the expression of Bcl-2 mRNA, with minimal impact on Bax mRNA expression. This comprehensive analysis of experimental data suggests that the cinnamic acid derivative 3,4,5-trihydroxycinnamate decyl ester has the ability to induce apoptosis in MCF-7 cells.

Research has shown that tamoxifen, a selective estrogen receptor modulator (SERM), has the potential to inhibit the development of breast cancer [78]. Motawi et al. [79] conducted a study to evaluate the combined effect of the cinnamic acid derivative caffeic acid phenethyl ester (CAPE) and tamoxifen on MCF-7 cells. The findings indicated that combining caffeic acid phenethyl ester (CAPE) with tamoxifen produced a synergistic cytotoxic effect on breast cancer cells and strongly induced apoptosis. This was evidenced by the downregulation of Bcl-2 and Beclin-1 protein levels. In essence, CAPE enhanced tamoxifen’s anti-tumor activity by promoting cancer cell apoptosis and reducing cell viability. Further experiments showed that the combination treatment increased caspase-3 activity and significantly elevated the tumor cell apoptosis rate, while also raising levels of glutathione and nitric oxide production. Additionally, the combination treatment significantly reduced the expression of Bcl-2, beclin-1, and VEGF genes within 24 to 48 h. Therefore, the joint application of CAPE and tamoxifen not only substantially enhances the cytotoxic effect but also activates apoptosis, downregulates proteins associated with cell survival, and inhibits tumor angiogenesis and metastasis through various mechanisms.

However, the combination of CAPE and tamoxifen carries potential risks of drug conflicts. For instance, CAPE may interfere with the metabolic activation of tamoxifen by inhibiting cytochrome P450 enzymes, and its antioxidant mechanisms could interfere with tamoxifen’s estrogen receptor modulation. Additionally, current data from in vitro cell models lack in vivo pharmacokinetic validation and clinical safety evidence. Therefore, clinical application requires systematic evaluation of drug interactions and long-term risks. In summary, cinnamic acid derivatives like CAPE possess the capability to influence pathways and proteins, thereby effectively inhibiting the growth and spread of breast cancer. However, the synergistic mechanisms and risks associated with their combination with traditional drugs still require further elucidation through rigorous clinical research.

Cinnamic acid derivatives have shown great potential in the treatment of breast cancer, providing valuable insights for therapy and drug development. Further research and exploration of these derivatives are expected to reveal more effective treatments, ultimately bringing greater hope and possibilities for patients with breast cancer.

### 6.2. Colorectal Cancer

Colorectal cancer (CRC) is a common type of cancer that impacts the gastrointestinal tract. It is the second highest cause of cancer-related deaths worldwide and ranks as the third most frequently diagnosed malignant tumor [76]. The early symptoms are not obvious [80], and its incidence rate is second only to lung cancer and gastric cancer in China [81]. While additional treatments such as surgery, radiation therapy, and chemotherapy can improve the survival rates of patients with colorectal cancer (CRC), leading to a 65% survival rate after 5 years, the 5-year survival rate for advanced CRC drops significantly to only 15% [82]. Complete surgical removal of the tumor and its metastatic sites improves the overall survival (OS) of patients with CRC. However, approximately 25% of CRC cases are diagnosed at an advanced stage with distant metastasis, which complicates surgical intervention. Therefore, there is a critical need to develop novel therapies for CRC.

Research has shown that histone deacetylation is a crucial component of epigenetic regulation and is a key contributor to tumorigenesis. Therefore, the inhibition of histone deacetylase (histone deacetylase, HDAC) can effectively hinder tumor growth [83,84]. Bingyan Zhu [9] investigated the impact of trans-cinnamic acid (t-CA) on histone deacetylase (HDAC) activity and found that t-CA exhibited an IC_50_ value of 250 µM for inhibiting HT29 cell proliferation. The compound inhibited cell growth in a dose-dependent manner and significantly induced apoptosis through the downregulation of HDAC I/II. Additionally, the study confirmed the effect of t-CA on histone acetylation in HT29 and MIA PaCa-2 cells. Further investigations involved treating HeLa cell nuclear extracts and HT29 cells with varying t-CA doses to assess HDAC I/II activity, revealing that t-CA directly inhibits HDAC activity. Subsequent experiments with different t-CA concentrations on HT29 and MIA PaCa-2 cells showed a dose-dependent increase in acetylated H3 and H4 proteins. The observed increase in acetylated H3 levels was consistent with the effects of the HDAC inhibitor TSA, suggesting that t-CA can function effectively as an HDAC inhibitor.

By investigating the impact of t-CA on apoptosis in HT29 and MIA PaCa-2 cells, the study found that t-CA increased Bax expression while decreasing PARP and Bcl-2 levels, indicating its ability to induce apoptosis in HT29 cells. In a HT29 colon cancer xenograft tumor model, t-CA doses of 1.0 and 1.5 mmol/kg inhibited tumor growth by 39% and 52.9% respectively. Pathological analysis of organs from t-CA treated mice showed no toxic changes, and no significant difference in body weight was observed compared to the control group, suggesting good tolerance to the administered dose of t-CA.

In summary, the research demonstrates that trans-cinnamic acid (t-CA) exerts an inhibitory effect on colon cancer tumors transplanted in nude mice. This anti-tumor activity of t-CA is likely mediated through the inhibition of histone deacetylase (HDAC) in the tumor cells. These findings offer a theoretical foundation and practical significance for the future development of novel therapeutic drugs.

### 6.3. Lung Cancer

Lung cancer is a complex and varied disease. The absence of reliable screening techniques frequently results in patients being identified at later stages, posing challenges for treatment [85]. Consequently, the prevalence of lung cancer has escalated, making it the primary cause of cancer-related mortality, with projections indicating a further increase in the future [86]. Within non-small cell lung cancer (NSCLC), LUAD is the most common subtype. While early-stage LUAD patients have a better prognosis, a notable percentage, ranging from 10% to 44%, still do not survive beyond a 5-year period post-surgical treatment [87,88]. At present, treatment modalities for LUAD encompass surgical resection, radiotherapy, chemotherapy, and immunotherapy, along with integrative approaches that combine Chinese and Western medicinal practices. Nevertheless, the frequently utilized anti-tumor agent cisplatin is notably dose-dependent. This drug is primarily metabolized in the liver and excreted through the kidneys [89], rendering it prone to side effects including hepatotoxicity and nephrotoxicity [90].

In order to investigate the cytotoxic effects of cinnamic acid on A549 cells, Gow-Chin Yen [10] conducted experiments using varying concentrations of both cis-cinnamic acid (c-CA) and trans-cinnamic acid (t-CA) in A549 cells. The IC_50_ values were determined to be approximately 180 µM for c-CA and 160 µM for t-CA after 48 h of treatment, indicating moderate cytotoxic potency against lung adenocarcinoma cells. Cytotoxicity was assessed using the MTT method. The study results showed a significant decrease in cell viability with doses exceeding 150 µM and a treatment duration of 48 h. Additionally, the research indicated that both c-CA and t-CA inhibited PMA-induced MMP-9 and MMP-2 activities in A549 cells. The experiment utilized PMA as an inducer to observe the inhibitory effects of c-CA and t-CA on A549 cells. Research findings indicate that at nontoxic doses, the inhibition of MMP-9 and MMP-2 by c-CA and t-CA is dose-dependent. After treatment, a significant decrease in the mobility of PMA-induced A549 cells was observed, with reductions of 64% and 63% in cell migration ability.

In summary, both c-CA and t-CA have demonstrated potential in inhibiting the invasion of A549 cells, effectively reducing cell migration and activities of MMP-2 and MMP-9, ultimately leading to the inhibition of tumor proliferation. This suggests that cinnamic acid could potentially be utilized as a drug to mitigate the invasion of lung adenocarcinoma, offering a solid theoretical foundation and practical significance for continued research into its molecular mechanisms and the development of new pharmaceuticals.

### 6.4. Prostate Cancer

Worldwide, prostate cancer ranks as the second most prevalent cancer among men, responsible for almost 400,000 deaths annually and constituting around 4% of all male cancer fatalities [91]. Prostate cancer is commonly linked to age, particularly affecting males aged 65 and older. Other factors such as dietary habits, obesity, genetic predisposition, and a history of sexually transmitted diseases may also contribute to the occurrence of prostate cancer [92]. The initial stages of prostate cancer are typically asymptomatic, which complicates early diagnosis. As the disease advances, patients may experience symptoms like frequent urination, difficulty urinating, increased nighttime urination, and back pain. Common treatments for prostate cancer include surgery, radiation therapy, chemotherapy, and androgen deprivation therapy, all of which can lead to adverse effects. For instance, androgen deprivation therapy may result in issues like erectile dysfunction, hot flashes, anemia, and depression [93]. Hence, there is a critical need to explore and develop new medications for the prevention and treatment of prostate cancer.

Masahiko Imai et al. [11] investigated the effect of the cinnamic acid derivative 3,4,5-trihydroxycinnamic acid decyl ester on the growth of prostate cancer PC-3 cells using the MTT method. Their experimental results indicated that at a concentration of 4 µM, this compound exhibited a 94% inhibitory effect on the growth of MCF-7 cells. Additionally, further studies revealed that 3,4,5-trihydroxycinnamic acid decyl ester could hinder the growth of cancer cells by modulating cell cycle distribution, enhancing caspase-3 activity, and influencing Bcl-2 mRNA expression. The experimental outcomes demonstrated that after administering this compound at varying concentrations, there was a significant increase in the number of cells in the G1 phase of PC-3 cells. This increase was positively correlated with the dosage of the drug. Concurrently, in treated cells, the number of cells in the G1 sub-phase displayed different degrees of increase at concentrations of 4 µM, 10 µM, and 20 µM, and the number of cells in the sub-G1 phase also showed a notable rise. These observations suggest that 3,4,5-trihydroxycinnamic acid decyl ester can promote apoptosis in PC-3 cells. Moreover, the study’s findings also indicated that this compound could significantly enhance the activity of caspase-3 in PC-3 cells while markedly decreasing the expression level of Bcl-2 mRNA. Therefore, it can be concluded that 3,4,5-trihydroxycinnamic acid decyl ester holds potential as an inducer of apoptosis in PC-3 cells.

In summary, in-depth research on the mechanism of cinnamic acid derivatives inducing apoptosis in prostate cancer cells not only offers valuable insights and avenues for exploring novel treatment strategies for prostate cancer but also provides guidance for the future use of cinnamic acid derivatives in its treatment.

### 6.5. Chronic Myeloid Leukemia

Chronic myeloid leukemia (CML) is a myeloproliferative neoplasm characterized by uncontrolled proliferation of mature myeloid cells [94]. The global incidence of adult leukemia is approximately 15% [95]. Despite individuals with CML being treated with Tyrosine kinase inhibitors (TKIs) having a life expectancy similar to the general population, managing the disease faces problems such as drug resistance and unknown causes of disease progression [96]. As the clinical benefits of traditional Chinese medicine gain recognition, an increasing number of active anti-tumor components from this medicine are being discovered, analyzed, and utilized. Cinnamic acid, in particular, has been extensively studied for its anticancer properties and may offer new directions and hope for CML therapy.

Münevver Yenigül et al. [12] investigated the impact of ethacrylic acid (EA) and cinnamic acid (CA) on chronic myeloid leukemia K562 cells over a period of 48 h. Cell viability was evaluated using the MTT assay to assess cytotoxicity. The findings indicated a dose-dependent decrease in cell proliferation with the administration of EA and CA. Individually, CA exhibited an IC_50_ of ~340 µM, whereas EA showed an IC_50_ around 90 µM for inhibiting K562 cell proliferation. Notably, the combination produced synergistic cytotoxicity, lowering cell viability by 63% at 300 µM CA (IC30) plus 100 µM EA. To further investigate the synergistic effect of EA and CA, an isowave plot test was performed. The findings revealed that when the concentration of EA was ≤100 µM, a combined administration with CA exhibited a synergistic cytotoxic effect on K562 cells. Annexin V-FITC/PI double staining was used to detect cell apoptosis and showed that the individual treatments with EA and CA increased the number of apoptotic cells, with the combined treatment yielding even more significant results. Flow cytometry analysis indicated a 36% reduction in the number of K562 cells in the G0/G1 phase upon combined treatment with EA and CA. Overall, these results suggest that the combined treatment with EA and CA significantly induces K562 cell apoptosis and leads to cell cycle arrest in the G2/M phase.

In summary, the synergistic effect of cinnamic acid with EA and CA has demonstrated a notable anti-proliferative impact on the K562 cell line, suggesting that combined application could be a promising treatment approach. These results offer an empirical foundation for utilizing cinnamic acid in chronic myeloid leukemia therapy, but further basic research is needed to verify its anti-tumor activity.

## 7. Anti-Diabetic

Diabetes is a chronic disease that urgently requires global attention, with its primary characteristic being the sustained elevation of blood glucose levels [97]. People with diabetes are affected by multi-organ system vascular disease, including microvascular and macrovascular complications [98], which may lead to renal or cardiovascular disease and increase the risk of myocardial infarction, stroke, and amputation, thereby increasing disability and mortality [99]. Although current drug treatments can effectively reduce high blood sugar, they cannot stably regulate daytime blood sugar levels and maintain them at optimal levels. In recent years, with the continuous development of dietary supplements and functional foods, the mechanism of phytochemical components in the prevention and treatment of diabetes and its complications has also received widespread attention. Among many bioactive substances, cinnamic acid and its derivatives, as compounds existing in nature, play an important role in the treatment of diabetes and its complications.

Rahman M Hafizur et al. [100] conducted a study to investigate the hypoglycemic effects of cinnamic acid in type II diabetic rats through acute experiments. The results showed that after oral administration of cinnamic acid, there was a significant reduction in blood sugar levels in diabetic rats within 2 h (*p* < 0.05), with the effect being both time- and dose-dependent. The OGTT method was used to confirm cinnamic acid’s ability to promote insulin secretion and demonstrate its anti-diabetic properties. The study found that blood glucose levels peaked within 45 min after glucose administration. Compared to the control group, oral administration of cinnamic acid reduced blood glucose levels by enhancing glucose tolerance and stimulating pancreatic beta cells to secrete insulin. Further experiments revealed that cinnamic acid also increased insulin secretion in in vitro studies in a concentration-dependent manner. It was observed that cinnamic acid significantly stimulated insulin secretion at a concentration of 50 μM (*p* < 0.01), with a more pronounced effect at a concentration of 100 μM (*p* < 0.001). Therefore, the study concluded that cinnamic acid can effectively lower blood glucose levels and improve glucose tolerance in diabetic rats by enhancing insulin secretion.

Chronic hyperglycemia in diabetes can lead to complications such as neuropathy, nephropathy, retinopathy, and increased cardiovascular disease risk [101]. An experiment by Hatice Gül Anlar et al. [6] investigated cinnamic acid (CA) for its protective effects against oxidative damage in blood, liver, and kidney cells caused by diabetes. Results showed reduced DNA damage in rats treated with CA compared to the diabetic control group, indicating CA’s strong DNA repair ability. The CA treatment group also had significantly higher insulin levels than the diabetic control group, supporting CA’s effectiveness in addressing diabetes-related changes. Moreover, CA treatment led to decreased levels of liver enzymes (ALT, AST, AP, GGT), suggesting a protective impact on liver function. Additionally, the CA group had lower levels of LDL, total cholesterol, and triglycerides compared to the diabetic control group, indicating CA’s potential in regulating blood lipids. Analysis of antioxidant enzyme activities revealed increased CAT, SOD, GSH-Px activities, and GSH levels in the plasma, liver, and kidney of the CA treatment group, indicating CA’s ability to enhance antioxidant defense mechanisms. In conclusion, cinnamic acid demonstrates notable protective effects on blood, liver, and kidney damage resulting from diabetes in rats, displaying promising potential for the treatment of diabetes and its complications.

To treat diabetic cardiomyopathy (DCM), Anupama Nair et al. [102] employed cinnamic acid (CA) as a cardioprotective agent against DCM damage. Male Wistar rats were subjected to a high-fat, high-fructose diet over a duration of 6 months and were administered a single dose of streptozotocin to induce a diabetes model. The findings revealed that diabetic rats exhibited insulin resistance and myocardial impairment, alongside significant elevations in total cholesterol, triglycerides, and low-density lipoprotein levels. The myocardial mass index, along with LDH, CKMB, ANP, and CRP levels, showed a notable increase in the diabetic group. Moreover, markers indicative of cardiac hypertrophy, such as TGF-β and β-MHC, were significantly elevated in diabetic rats. Furthermore, the study disclosed that pro-inflammatory cytokines (TNF-α, IL-6) and serum lipid peroxide levels were markedly increased in diabetic rats. Histopathological examinations indicated the presence of inflammation and necrosis within the hearts of the rats. Following CA treatment, there was alleviation in insulin resistance, normalization of blood lipid levels, and reduction in pro-inflammatory factors. Consequently, oral administration of CA has the potential to inhibit the progression of DCM through its cardioprotective, anti-inflammatory, anti-dyslipidemic, and anti-diabetic effects. In turn, CA maintains the integrity of the heart by preventing myocardial hypertrophy and fibrosis.

In summary, cinnamic acid has been shown to have therapeutic benefits for diabetes and its complications, suggesting potential avenues for the treatment of the disease. This finding offers valuable insights for future research on utilizing cinnamic acid for diabetes management.

## 8. Anti-Depressant

Depression is a serious psychiatric disorder characterized by persistent low mood, psychomotor retardation, anhedonia (loss of interest), low self-esteem, and sleep disturbances [103]; it is a major public health concern. Notably, cinnamic acid and some of its derivatives have exhibited antidepressant-like effects in preclinical studies, possibly by reducing neuroinflammation and modulating neurotransmitter systems [104]. Currently, commonly utilized antidepressants encompass tricyclic antidepressants (e.g., amitriptyline, nortriptyline, and demipramine), selective serotonin reuptake inhibitors (SSRIs, such as fluoxetine, sertraline, and citalopram), and monoamine oxidase inhibitors (MAOIs, like moclobemide) [105]. While these antidepressants prove effective for a majority of patients, some individuals fail to achieve the desired outcomes. Moreover, these medications are linked to various adverse effects and may induce emotional blunting in patients [106]. Thus, it is essential to discover new, safer, and more efficacious medications for the treatment of depression. Lately, natural products exhibiting antidepressant properties have emerged as a significant research focus and could potentially become a key source of antidepressant drugs.

In a study conducted by Rengong Zhuo et al. [107], it was found that cinnamic acid (CA) has the potential to alleviate depression-like behavior in mice induced by lipopolysaccharide (LPS) by dampening pro-inflammatory responses and enhancing oxidative stress levels. The experiment utilized the sucrose preference test (SPT), forced swimming test (FST), and tail suspension test (TST) to assess depressive-like behavior, while also measuring interleukin-6 (IL-6), tumor necrosis factor-α (TNF-α), superoxide dismutase (SOD), glutathione, malondialdehyde (MDA), and brain-derived neurotrophic factor (BDNF) levels in the hippocampus and cortical tissue of mice. The results revealed that doses of 100 mg/kg and 200 mg/kg of CA were effective in reducing depressive-like behaviors induced by TST, FST, and SPT, while also increasing the expression levels of IL-6 and TNF-α in the hippocampus and cortex, as well as improving the oxidative stress parameters SOD, glutathione, and MDA. Furthermore, the study indicated that CA significantly reversed the LPS-induced decrease in brain-derived neurotrophic factor (BDNF) levels in the hippocampus and cortex of mice.

In summary, CA has an antidepressant effect on LPS-induced mice and significantly improves BDNF injury. Therefore, CA can be used as a promising antidepressant treatment drug.

## 9. Other Pharmacological Effects

Neuroprotection refers to the process of maintaining or restoring neural functions by mitigating or preventing structural and functional damage to neurons caused by injury, disease, or aging. This is primarily achieved through various molecular mechanisms, including anti-inflammatory, antioxidant, anti-apoptotic, regulation of autophagy, modulation of calcium ion balance, and improvement of mitochondrial function [108]. In diseases such as Alzheimer’s and cerebral ischemic injury, cinnamic acid derivatives demonstrate significant neuroprotective effects by enhancing mitochondrial function, thereby effectively slowing the progression of pathology. Dmitry I Pozdnyakov et al. [109,110] found that 4-hydroxy-3,5-di-tert-butylcinnamic acid (ATACL) improves memory deficits and provides neuroprotection in rats with Alzheimer’s disease (AD) induced by Aβ1-42 injection, and in those with cerebral ischemic injury. Furthermore, in AD rats, ATACL reduces the expression of p-Tau protein in the hippocampus, restores mitochondrial function by enhancing ATP production, respiratory rate, and glycolytic capacity, while also decreasing intracellular calcium, caspase-3, and H2O2 levels. In rats with cerebral ischemic injury, ATACL increases the levels of citrate synthase, superoxide dismutase (SOD), catalase, and glutathione peroxidase (GPx) in the hippocampal region, while reducing the concentrations of apoptosis-inducing factor (AIF) and caspase-3. These findings indicate that ATACL exerts neuroprotective effects by improving mitochondrial function through multiple pathways, enhancing antioxidant capacity, and inhibiting apoptosis, thereby providing a novel potential strategy for the treatment of neurological diseases. Free radicals are the primary cause of various degenerative conditions, such as diminished cardiac systolic function, lumbar disc herniation, and other chronic illnesses [111]. Despite the presence of antioxidant defense and repair mechanisms in organisms, they are not enough to completely prevent cellular damage. As a result, antioxidants are utilized to delay or mitigate the impact of oxidative damage [112]. Cinnamic acid, a naturally occurring white crystalline organic acid found in plants, was administered in a study by Esmaeel Babaeenezhad et al. [113]; the findings indicated that cinnamic acid improved oxidative stress, subsequently alleviating rat nephrotoxicity and reducing transaminase activity. Therefore, cinnamic acid demonstrates significant antioxidant properties in biological systems.

Globally, liver disease has become a significant issue threatening public health, and given the frequent occurrence of complications during the disease progression, existing treatment methods remain inadequate. In this context, Liseth Rubí Aldaba-Muruato et al. [114] investigated the protective effects of the cinnamic acid derivative 3-(4-Phenoxy)phenyl-N-[(3,4-dichlorophenyl)methyl]prop-2-enamide(LQM755) in a carbon tetrachloride-induced acute liver injury model using Wistar rats. Experimental data indicate that LQM755 can effectively ameliorate pathological damage in liver tissue, and reduce levels of ALT, ALP, GGT, total bilirubin, and direct bilirubin, thereby improving liver function. This demonstrates that LQM755 has significant hepatoprotective effects against carbon tetrachloride-induced acute liver injury, providing crucial experimental evidence for the development of novel therapeutic drugs for liver diseases.

Obesity induces metabolic disorders, which subsequently lead to a spectrum of diseases including hyperlipidemia, diabetes, hypertension, and atherosclerosis [115]. Kais Mnafgui et al. [116] conducted a study to assess the anti-obesity effects of cinnamic acid (CA) in rats fed a high-fat diet (HFD). The findings indicated that the body weight of rats in the HFD group increased by approximately 27%, coupled with a significant 103% rise in serum lipase activity. Concurrently, there was an elevation in serum total cholesterol (T-Ch), triglycerides (TG), and low-density lipoprotein cholesterol (LDL-cholesterol) levels. Additionally, the activity of the angiotensin-converting enzyme (ACE) in the serum of HFD-fed rats showed a significant increase. Following treatment with CA, the blood lipid levels of HFD-fed rats returned to normal, and the activities of both lipase and ACE were restored to standard levels. Echocardiographic assessments revealed that CA could enhance the diameter of the aorta and aortic arch, thereby preventing vasoconstriction while simultaneously ameliorating hepatic steatosis and nephrotoxicity indicators. Consequently, cinnamic acid demonstrates anti-obesity effects by inhibiting the activities of lipid-digesting enzymes and ACE.

Asthma is a chronic immunological condition characterized primarily by inflammation and hyperresponsiveness in the airways [117]. Approximately 300 million people worldwide currently suffer from asthma [118]. Research suggests that genetic factors and social pressures contribute to the development and worsening of asthma, leading to higher rates of morbidity and mortality [119]. Cinnamic acid has been shown to effectively reduce leukocyte infiltration and suppress pro-inflammatory cytokine production, thus mitigating the inflammatory response and tissue damage in the lungs of asthma patients [120], providing a new direction for the prevention and treatment of asthma.

## 10. Toxicological Evaluation

Cinnamic acid and its derivatives generally exhibit a favorable safety profile, although their toxicity levels can vary depending on dose, route of administration, and specific chemical modifications. Most in vitro and in vivo studies indicate relatively low acute and sub-chronic toxicity within conventional dose ranges, making these compounds widely employed in food, cosmetics, and pharmaceuticals. Nevertheless, certain derivatives at high doses or administered via specific routes may induce adverse reactions, warranting cautious evaluation in clinical and industrial settings [13].

Acute toxicity studies commonly utilize the median lethal dose (LD_50_) to assess safety margins. For example, oral administration of cinnamic acid up to ~2000 mg/kg body weight in animals caused only mild adverse effects, indicating a broad safety margin in vivo [121]. By contrast, in vitro studies showed that concentrations above 500 μM can collapse the mitochondrial membrane potential, trigger a burst of reactive oxygen species (ROS), and induce apoptosis in hepatocytes. Additionally, more lipophilic cinnamic acid derivatives were about five times more toxic to skin cells than the parent compound, and halogenated derivatives activated caspase-3–mediated apoptosis in renal tubular cells at around 100 μM. Additionally, these compounds demonstrate a biphasic dose response: at low doses (<50 μM), they activate the Nrf2 pathway to confer antioxidant protection, whereas at high doses (>200 μM), they inhibit superoxide dismutase (SOD) activity and trigger ferroptosis. Particularly in metabolic disease models, the antioxidant–pro-oxidant switch threshold decreases by 60%, exacerbating organ damage risks. Therefore, traditional LD50 assessment has limitations and requires the integration of a “three-track verification strategy” that combines animal LD50 testing, cellular mitochondrial function detection, and organ-specific biomarker analysis, along with the optimization of dosing regimens through targeted delivery systems, to precisely control dose-dependent toxicity risks.

Many sub-chronic and chronic toxicity studies focus on changes in liver and kidney function, given that these organs play critical roles in metabolizing and excreting exogenous substances. Under high-dose exposure, cinnamic acid derivatives can induce significant hepatotoxicity, manifested by a sharp increase in serum transaminase (ALT, AST) levels, which is directly related to the metabolic accumulation of the compounds in the liver. Simultaneously, it may cause renal tubular epithelial damage, interfering with urine production and excretion functions. However, this organ toxicity shows a clear dose dependency. At medium doses, most cinnamic acid derivatives only cause slight and reversible alterations in organ structure or biochemical markers, such as a mild elevation of serum transaminases (ALT, AST) or minor changes in renal tubular epithelium [19]. The clinical therapeutic doses have undergone rigorous safety assessments, posing a minimal risk of toxicity. For derivatives containing multiple hydroxyl groups or increased lipophilicity, additional attention should be given to potential cytotoxicity or immune responses at high doses [122]. Furthermore, since cinnamic acid is an aromatic compound, some individuals may be allergic to it or its derivatives, leading to localized allergic reactions or contact dermatitis, especially when these derivatives are incorporated into cosmetics and personal care products. It is advisable to conduct patch tests [123]. Generally, these adverse reactions usually subside or resolve after discontinuation of use.

Regarding genotoxicity and carcinogenicity, most studies have not identified any clear mutagenic or oncogenic risks with cinnamic acid and its common derivatives at standard doses. Some investigations have even suggested that caffeic acid esters and multi-hydroxyl-substituted derivatives could possess cancer-inhibitory properties, such as inducing apoptosis or reducing oxidative stress [124]. Nonetheless, further large-scale, long-term research—especially in transgenic animal models—is needed to rule out potential hazards, particularly when high doses or prolonged intake are involved.

Clinically and nutritionally, cinnamic acid derivatives are believed to offer a relatively wide therapeutic window. In preclinical studies, animals are typically monitored closely for liver and kidney function, biochemical indices, and immune responses to ensure safety. Because cinnamic acid is found extensively in nature and in daily diets, several derivatives have been granted Generally Recognized as Safe (GRAS) status [19,125]. However, increasing their concentration in food products or applying nano-based formulations still calls for adherence to regulatory guidelines and systematic toxicological assessment.

With the advancement in nanotechnology, cinnamic acid derivatives are increasingly being incorporated into nanocarriers and nanoemulsions to improve their solubility and bioavailability. However, such nanoscale formulations may introduce new safety concerns (e.g., unexpected tissue accumulation or immune system dysregulation). Consequently, research into nano-formulated cinnamic acid derivatives must carefully balance improved efficacy with long-term safety considerations. Overall, comprehensive toxicological evaluations of cinnamic acid and its derivatives—across various delivery methods, patient populations, and disease conditions—remain essential before broader clinical adoption and industrial use.

## 11. Current Application Status and Development Prospects

Numerous studies have shown that cinnamic acid and its natural derivatives (from cinnamon bark/twig) possess a broad spectrum of pharmacological activities, including anti-inflammatory and anti-tumor effects. They have demonstrated significant therapeutic potential against infections (bacterial and fungal) and other diseases. In medicine, cinnamic acid has been used to treat superficial fungal skin infections and even as a dietary supplement for metabolic health. Certain derivatives have found niche clinical uses; for example, kojic acid monomethoxycinnamate is used adjunctively for solar dermatitis. In the cosmetics industry, octyl methoxycinnamate is a key UVB-filtering ingredient in sunscreens, the combination of ferulic acid (a cinnamic derivative) with vitamin C produces skin-whitening effects, and cinnamyl alcohol serves as both a fragrance and a preservative. Given these extensive benefits, cinnamic acid and its derivatives show considerable promise as natural health products. Synthetic derivatives of cinnamic acid with varied structures can be synthesized through chemical synthesis or biological transformation techniques, offering enhanced medicinal efficacy and reduced side effects. In terms of antibacterial activity, the combination of cloxacillin with cinnamic acid derivative octanoic acid can reduce its minimum inhibitory concentration and significantly enhance the anti-biofilm effect [126]. In-depth research into the pharmacological mechanisms of cinnamic acid, along with the evaluation of its derivatives’ pharmacological activities, could not only improve the efficacy of cinnamic acid but also provide a stronger foundation for its clinical applications. Recent studies suggest that nanomedicines, as innovative drug delivery systems, can greatly enhance the stability, solubility, and bioavailability of drugs. Nanoparticles formed through the self-assembly of berberine and cinnamic acid do not necessitate any special treatment during this process. These nanoparticles exhibit significantly greater efficacy than first-line antibiotics such as norfloxacin, amoxicillin, and tetracycline in inhibiting multidrug-resistant *Staphylococcus aureus*. Furthermore, the combination of cinnamic acid and mangiferin presents a promising avenue for the development of a new therapeutic agent for the management of rheumatoid arthritis. Progress in developing nanomedicines incorporating cinnamic acid and its derivatives could lead to better-controlled drug release, thereby improving both efficacy and safety of treatments. Therefore, it is crucial for future research to intensify the development and application of cinnamic acid to meet growing market demands and significantly contribute to human health. To systematically compare these derivatives, Table 3 summarizes their key physicochemical properties and general activities. Considering the different experimental contexts, the pharmacological studies have been further classified clearly into separate tables: Table 4 summarizes in vitro studies, Table 5 summarizes animal (in vivo) studies, and Table 6 specifically highlights clinical studies. Each table includes detailed information regarding experimental models, dosages, treatment durations, and observed pharmacological outcomes, aiming to improve clarity and facilitate direct comparisons across different research contexts.

## 12. Summary

Cinnamic acid (CA), a crucial active ingredient in traditional medicinal plants, demonstrates significant scientific research value and industrial potential in modern pharmaceutical innovation. Extensive studies have confirmed that this compound and its derivatives possess core pharmacological activities, including anti-inflammatory and anti-tumor effects. By applying innovative methods such as structural modification and nanotechnology, researchers have successfully overcome limitations of natural compounds, enabling breakthrough applications in pharmaceuticals and cosmetics. Notably, CA derivatives designed for major diseases like cancer, neurodegenerative disorders, and metabolic diseases exhibit highly promising clinical translation prospects. Current research focuses on elucidating structure–activity relationships, developing high-activity derivatives, and advancing nano-drug delivery systems based on CA derivatives. These strategies address critical bottlenecks such as low bioavailability and inadequate targeting, thereby significantly enhancing therapeutic efficacy while reducing adverse effects. Multiple preclinical and early-stage clinical studies are systematically evaluating the safety and efficacy of CA derivatives, laying the groundwork for subsequent translation into practice. To accelerate innovation, establishing an industry–academia–research collaborative mechanism is imperative, effectively integrating multidisciplinary resources and optimizing the entire chain from basic research to industrialization. Future high-potential CA derivative candidates, developed through structural optimization and advanced formulation technologies, are poised to become strategic resources in the healthcare industry. These innovations will not only deliver safer, more efficient solutions for disease prevention and health management but also enable a rapid transition from laboratory research to clinical application, ultimately creating greater value in medicine and healthcare.

## 13. VOSviewer

**Figure 5:**
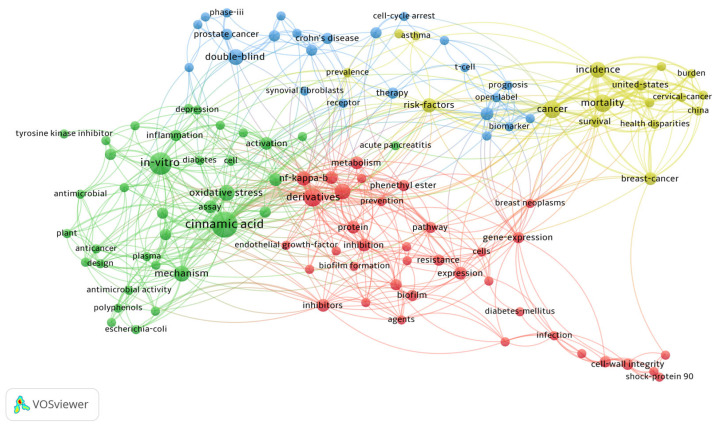
VOSviewer of CA and derivatives.

We used VOSviewer (version 1.6.20) to create a figure highlighting cinnamic acid and derivatives themes, activities, and sources to enhance the review methodology. Each node represents a keyword, with node size indicating the keyword’s importance within the literature. The lines connecting nodes represent co-occurrence relationships between keywords. Keywords are color-coded based on the clusters they belong to, where keywords in the same cluster are more likely to co-occur in the literature (Figure 5).

## Figures and Tables

**Table 1:** Physical and chemical properties of cinnamic acid and its derivatives.

Compound	Molecular Weight	pKa	Notable Substituents/Configuration	Water Solubility	Key Properties
Cinnamic acid (CA)	148.16	~4.44	Basic scaffold: benzene ring + propenoic	0.46–0.50 g/L (25 degrees)	Parent compound, moderate acidity, forms basis for multiple derivatives
Trans-cinnamic acid (t-CA)	148.16	~4.44	Trans config around C=C	~0.47 g/L (25 degrees)	More stable vs. cis isomer, reported HDAC inhibition, anticancer activities
Caffeic acid phenethyl ester (CAPE)	284.29	~4.25	Additional –OH groups on ring + phenethyl group	Very low (~0.1 mg/mL)	Potent antioxidant, anti-inflammatory, anti-tumor; poor water solubility
3,4,5-trihydroxycinnamic acid decyl ester	338.37	~4.0	3 –OH groups on benzene + decyl ester	Very low	Enhanced lipophilicity, significant cytotoxic effects in some cancer cell lines
p-Coumaric acid (4-hydroxycinnamic acid)	164.16	~4.34	Hydroxy at para-position	2.5 g/L (25 degrees)	Greater hydrophilicity vs. cinnamic acid, antioxidant and anti-inflammatory activities

**Table 2:** Summary of main cinnamic acid derivatives, their structural motifs, molecular targets, and clinical significance.

Derivative	Key Structural Motif	Main Molecular Targets	Reported Clinical/Preclinical Significance
Caffeic acid phenethyl ester (CAPE)	2 phenolic –OH + phenethyl tail	NF-κB, VEGF, COX-2	Synergizes with tamoxifen in ER^+^ breast cancer; anti-inflammatory
Trans-cinnamic acid (t-CA)	Trans-C=C	HDAC I/II, MMP-2/-9	HDAC-dependent apoptosis in CRC; anti-invasive in NSCLC
Cis-cinnamic acid (c-CA)	Cis-C=C	MMP-2/-9	Co-inhibits invasion with t-CA in NSCLC
3,4,5-Trihydroxy-cinnamic acid decyl ester	Tri-OH ring + decyl ester	Caspase-3, Bax/Bcl-2	Potent apoptosis in breast and prostate cancer cells
Methyl caffeate	Methoxy + catechol	Ergosterol biosynthesis (fungi)	Inhibits *Candida albicans* growth
Methyl 2-nitrocinnamate	Nitro substitution	Ergosterol biosynthesis	Synergistic antifungal against *C. albicans*
p-Coumaric acid	Para–OH	NF-κB, MAPK	Antioxidant/anti-inflammatory prototype

**Table 3:** Pharmacological effects and potential mechanisms of action exhibited by cinnamon acid against different characteristics.

Pharmacological Effects	Aimed Subtype	Main Targets	Potential Mechanisms	Reference
Anti-breast cancer	Breast cancer	MCF-7 cells	Inhibition of cell growth, cell cycle arrest, apoptosis induction via caspase-3 activation, downregulation of Bcl-2 mRNA	Masahiko Imai et al. [11], Motawi et al. [79]
Anti-colorectal cancer	Colorectal cancer (CRC)	HT29, MIA PaCa-2 cells	HDAC inhibition, increased acetylated H3 and H4 proteins, apoptosis induction through Bax expression, reduced PARP and Bcl-2	Bingyan Zhu [9]
Anti-lung cancer	Non-small cell lung cancer (NSCLC)	A549 cells	Inhibition of cell viability, reduction in MMP-2 and MMP-9 activities, decreased cell migration	Gow-Chin Yen [10]
Anti-prostate cancer	Prostate cancer	PC-3 cells	Inhibition of cell growth, cell cycle arrest, apoptosis induction via caspase-3 activation, decreased Bcl-2 mRNA	Masahiko Imai et al. [11]
Anti-chronic myeloid leukemia	Chronic myeloid leukemia (CML)	K562 cells	Cytotoxic effect, cell cycle arrest, apoptosis induction, synergistic effect with ethacrylic acid	Münevver Yenigül et al. [12]
Anti-acute pancreatitis	Acute pancreatitis	Rats with acute pancreatitis	Reduction of oxidative stress, inhibition of ERK1/2, JNK, and p38 in MAPK signaling pathways, downregulation of NF-κB and NLRP3, reduced apoptosis via caspase activation	Omayma AR Abozaid [25]
Anti-acute hepatitis	Acute hepatitis	Rats with acute hepatitis	Reduction of serum ALT, AST, and ALP activities, decrease in TNF-α, IL-1β, and IL-18 levels, inhibition of TLR4 and MyD88, reduced NLRP3 and NF-κB, decreased apoptosis	Ehab A. Ibrahim et al. [28]
Anti-colitis	Ulcerative colitis, colitis	Mice with DSS-induced colitis, rats with DNBS-induced colitis	Reduction in MDA levels, increase in SOD and CAT levels, activation of GPR109A, reduction in MPO activity, inflammatory mediators, and IL-10 levels	Maysam A Hussein et al. [4], Changyu Kang et al. [35]
Anti-rheumatoid arthritis	Rheumatoid arthritis	AIA-M rat models	Inhibition of TLR4/PI3K/AKT/NFkB/NLRP3 pathway, reduction in inflammatory cytokines TNF-a, IL-6, IL-12, decreased caspase-1 expression, reduced IL-1β release	Weijie Li [41]
Anti-periodontitis	Periodontitis	Wistar rats with ligation-induced periodontitis	Decrease in RANKL expression, inflammation, osteoclast count, increase in OPG expression, osteoblast count, reduction in PPAR-γ and COX-2 levels	Ozkan Karatas [46]
Antibacterial effect	*Staphylococcus aureus*	MRSA	Inhibition of bacterial growth, biofilm formation, cell membrane damage, and nutrient intake; enhanced antibacterial activity with CA-BBR NPs	Huang Xuemei et al. [53]
Antibacterial effect	*Pseudomonas aeruginosa*	PAO1 strain	Reduction in exovirulence factor production, biofilm formation, cell surface hydrophobicity, exogenous DNA, and EPS; decreased biofilm thickness	Rajkumari et al. [61]
Antibacterial effect	Foodborne Pseudomonas	Psychrophilic Pseudomonas	Disruption of cell membrane homeostasis, reduced ATPase activity, cell membrane depolarization, decreased intracellular pH, increased bacterial mortality	Yuxiang Zhang et al. [16]
Anti-diabetic	Type II diabetes	Diabetic rats	Reduction in blood glucose levels, improved glucose tolerance, enhanced insulin secretion, reduced oxidative stress, decreased liver enzyme levels	Rahman M Hafizur et al. [100], Hatice Gül Anlar et al. [6], Anupama Nair et al. [102]
Anti-depressant	Depression	LPS-induced mice	Reduction in depressive-like behaviors, dampening pro-inflammatory responses, enhancing oxidative stress parameters, reversing decrease in BDNF levels	Rengong Zhuo et al. [107]
Antioxidant	Nephrotoxicity	Rats	Improved oxidative stress, reduced transaminase activity	Esmaeel Babaeenezhad et al. [113]
Anti-obesity	Obesity	HFD-fed rats	Normalization of blood lipid levels, reduced lipase, and ACE activities enhanced aortic diameter, prevention of vasoconstriction, amelioration of hepatic steatosis	Kais Mnafgui et al. [116]
Anti-asthma	Asthma	Asthma patients	Reduction in leukocyte infiltration, suppression of pro-inflammatory cytokine production, mitigation of inflammatory response and tissue damage in the lungs	Haidar AL-Saffar et al. [120]

**Table 4:** In vivo experimental parameters of cinnamic acid and its derivatives in different disease models.

Chemical Compound	Model	Duration	Dose	Reference
Cinnamic acid nanoparticles (CA-NPs)	L-arginine- and gamma ray-induced acute pancreatitis rat model	21 d	60 mg/kg	Omayma AR Abozaid’s [25]
	D-galactosamine and gamma radiation-induced acute hepatitis rat model	30 d	60 mg/kg	Ehab A. Ibrahim et al. [28]
Cinnamic acid (CA)	Mouse model of ulcerative colitis induced by dextran sodium sulfate	7 d	25~50 mg/kg	Maysam A Hussein et al. [4]
	Induction of periodontitis rat model by ligating the first mandibular tooth around the left and right mandibles with 4-0 silk thread	30 d	7 mg/kg	Ozkan Karatas [46]
	STZ-induced non-obese type II diabetes mellitus rat model	60 min	5~10 mg/kg	Rahman M Hafizur et al. [100]
	Rat model of type 1 diabetes induced by streptozotocin (STZ)	28 d	50 mg/kg	Hatice Gül Anlar et al. [6]
	High-fat high-fructose and streptozotocin-induced diabetes rat model	60 d	5~10 mg/kg	Anupama Nair et al. [102]
	LPS-induced depression mouse model	14 d	100~200 mg/kg	Rengong Zhuo et al. [107]
	Rats on a high-fat diet	7 weeks	30 mg/kg	Kais Mnafgui et al. [116]
trans-Cinnamic acid (t-CA)	2,4-dinitrobenzenesulfonic acid (DNBS) induced colitis rat model	6 d	15~30 mg/kg	Changyu Kang et al. [35]
Cinnamic acid and mangiferin	AIA-M rat models	30 d	46.652 mg/kg cinnamic acid 600.912 mg/kg mangiferin	Weijie Li [41]
Synthesized cinnamic acid and berberine into organic nanostructures (CA-BBR NPs)	Zebrafish larvae	72 h	2.5~80 μM	Huang Xuemei et al. [53]
4-Hydroxy-3,5-di-tret-butyl cinnamic acid	Brain ischemia in rats	3 d	25~100 mg/kg	Dmitry I Pozdnyakov et al. [110]
	Aβ1-42-induced AD rat model.	60 d	100 mg/kg	Dmitry I Pozdnyakov et al. [109]
3-(4-Phenoxy)phenyl-N-[(3,4-dichlorophenyl)methyl]prop-2-enamide	CCl4-induced acute liver injury rat model.	2 d	20 mg/kg	Liseth Rubí Aldaba-Muruato et al. [114]

**Table 5:** In vitro experimental parameters of cinnamic acid and its derivatives in different disease models.

**Chemical Compound**	**Model**	**Duration**	**Dose**	**Reference**
Synthesized cinnamic acid and berberine into organic nanostructures (CA-BBR NPs)	Multidrug-resistant *S. aureus* Madin–Darby canine kidney (MDCK) cells	16 h 24–48 h	0.0325~0.2 μmol/mL 1.5625~50 μM	Huang Xuemei et al. [53]
Cinnamic acid (CA)	Strain, *P. aeruginosa* PAO1	24 h	100~500 μg/mL	Rajkumari et al. [61]
	*Pseudomonas fragi* 38-8	30 min	0~1 mg/mL	Yuxiang Zhang et al. [16]
Ferulic acid and caprylic acid	Wild-type *P. aeruginosa* PA14 Wild-type *P. aeruginosa* PAO1 Biosensor strain *P. aeruginosa* PAO1 pqsA CTXluxHpqsA Reporter strain PA14-R3 developed by Massai et al.	0–24 h	6.25~1000 µg mL^−1^	Ariana S.C. Gonçalves et al. [62]
Methyl caffeate and methyl 2-nitro cinnamate	*Candida albicans* ATCC-76645, LM-106, LM-23		128~256 μg/mL	Tamires C. Lima et al. [70]
Chloro-α-methylcinnamic acid 4-methylcinnamic acid	*Aspergillus fumigatus*	5~7 d	0.1~1.0 mM	Jong H. Kim et al. [71]
3,4,5-trihydroxycinnamate decyl ester	MCF-7 breast cancer cells	72 h	0.4~20 µM	Masahiko Imai et al. [11]
	Prostate cancer PC-3 cells			
Caffeic acid phenethyl ester (CAPE)	MCF-7 breast cancer cells	48 h	0.1~200 mM	Motawi et al. [79]
trans-Cinnamic acid (t-CA)	HT-29 human colon cancer cells MIA PaCa-2 human pancreatic cancer cells	48 h	0.09~2.72 mM	Bingyan Zhu [9]
cis-Cinnamic acid (c-CA) and trans-cinnamic acid (t-CA)	A549 human lung adenocarcinoma cells	24–48 h	0~200 μM	Gow-Chin Yen [10]
Ethacrylic acid (EA) and cinnamic acid (CA)	K562 chronic myeloid leukemia cells	48 h	50~500 µM	Münevver Yenigül et al. [12]

**Table 6:** Summary of clinical studies investigating cinnamic acid and its derivatives.

Compound	Clinical Condition	Dose	Duration	Clinical Outcome/Effects	Reference
Cinnamic acid	Asthma patients	200 mg/day	4 weeks	Improved pulmonary function, reduced inflammatory markers	Haidar AL-Saffar et al. [120]
CAPE	Breast cancer patients	20 mg/day (combined with Tamoxifen)	3 months	Enhanced apoptotic activity, reduced angiogenesis markers (VEGF), potential risk of drug interaction noted	Motawi et al. [79]

## Data Availability

No new data were created or analyzed in this study. Data sharing is not applicable.

## Abbreviations

ACE	angiotensin-converting enzyme	LB	Luria–Bertani
AD	Alzheimer’s disease	LD50	median lethal dose
AH	Acute hepatitis	LDH	Lactate dehydrogenase
AIA-M	adjuvant-induced arthritis-modified rat model	LDL	low-density lipoprotein
AIF	apoptosis-inducing factor	LPS	Lipopolysaccharide
ALP	Alkaline phosphatase	LUAD	Lung Adenocarcinoma
ALT	Alanine aminotransferase	LQM755	3-(4-phenoxy)phenyl-N-[(3,4-dichlorophenyl)methyl]prop-2-enamide
ANP	Atrial Natriuretic Peptide	MAOIs	monoamine oxidase inhibitors
AP	alkaline phosphatase	MAPK	Mitogen-activated protein kinase
ASK1	Apoptosis Signal-regulating Kinase 1	MDA	malondialdehyde
AST	Aspartate aminotransferase	MDCK	Madin-Darby canine kidney
ATACL	4-hydroxy-3,5-di-tert-butylcinnamic acid	MG	mangiferin
ATP	Adenosine Triphosphate	MIC	minimum inhibitory concentration
Aβ	Bate-Amyloid	Micro-CT	Quantitative micro-computed tomography
BAX	BCL-2-associated X protein	MMP-2	Matrix metalloproteinase-2
BBR	berberine	MMP-9	Matrix metalloproteinase-9
Bcl-2	B-cell lymphoma-2	MPO	Myeloperoxidase
BDNF	brain-derived neurotrophic factor	MPZ	mepenzolate
BMD	bone mineral density	MRSA	Methicillin-resistant *Staphylococcus aureus*
BV/TV	bone volume/tissue volume	MTP	Microtitre plates
CA	Cinnamic acid	MTT	3-(4,5-dimethylthiazol-2-yl)-2,5-diphenyltetrazolium bromide
CA-NPs	cinnamic acid nanoparticles	MTX	methotrexate
CAPE	caffeic acid phenethyl ester	MyD88	Myeloid Differentiation Primary Response 88
CAT	catalase	NF-κB	Nuclear factor kappa-light-chain-enhancer of activated B cells
c-CA	cis-Cinnamic acid	NLRP3	NOD-like receptor protein 3
CINC-3	neutrophil chemoattractant-3	NPs	nanoparticles
CKMB	Creatine Kinase-MB	NSAIDs	nonsteroidal anti-inflammatory drugs
CLSM	confocal laser scanning microscopic	NSCLC	non-small cell lung cancer
CML	Chronic myeloid leukemia	Nrf2	Nuclear factor erythroid 2-related factor 2
COX-2	Cyclooxygenase-2	–OCH3	methoxy
CRC	Colorectal cancer	OGTT	oral glucose tolerance test
CRP	C-Reactive Protein	–OH	hydroxy
CSH	cell surface hydrophobicity	OPG	osteoprotegerin
DCM	diabetic cardiomyopathy	OS	overall surviva
D-Gal	d-galactosamine	p38	p38 mitogen-activated protein kinase
DMARDs	disease-induced anti-rheumarthritis drugs	PA	*Pseudomonas aeruginosa*
DNBS	dinitrobenzene sulfonic acid	PARP	Poly ADP-ribose polymerase
DSS	dextran sodium sulfate	PI3K	Phosphatidylinositol 3-kinase
EA	ethacrylic acid	PMA	phorbol-12-myristate-13-acetat
eDNA	exogenous DNA	PPAR-γ	Peroxisome proliferator–activated receptor-γ
EPS	polysaccharide substances in *Pseudomonas aeruginosa*	QS	quorum sensing
ERK1/2	extracellular signal-regulated kinase 1/2	RA	Rheumatoid arthritis
FESEM	field-emission scanning electron microscopy	RANKL	receptor activator of nuclear factor κ-B
FLS	fibroblast-like synoviocytes	SEM	scanning electron microscopy
FST	forced swimming test	SERM	selective estrogen receptor modulator
GGT	gamma-glutamyl transferase	SOD	superoxide dismutase
GPR109A	G-protein-coupled receptor 109A	Sp	separation
GPx	glutathione peroxidase	SPT	sucrose preference test
GRAS	Generally Recognized as Safe	SSRIs	selective serotonin reuptake inhibitors
GSH	total glutathione	Tb	trabecular
GSH-Px	glutathione peroxidase	t-CA	trans-Cinnamic acid
GSSG	oxidized glutathione	tCA-GA	trans-Cinnamic acid and synthesized its conjugates with glutamic acid
H&E	hematoxylin and eosin	T-Ch	total cholesterol
HDAC	histone deacetylase	TEM	transmission electron microscop
HFD	high-fat diet	TG	triglycerides
IBD	Inflammatory bowel disease	TGF-β	Transforming growth factor-beta
IC30	Inhibitory Concentration for 30% effect	Th	thickness
IC50	half maximal inhibitory concentration	TKIs	Tyrosine kinase inhibitors
IL-10	Interleukin-10	TLR4	Toll-like receptor 4
IL-12	Interleukin-12	TMD	tissue mineral density
IL-16	Interleukin-16	TNF-α	Tumor necrosis factor alpha
IL-18	Interleukin-18	TSA	trichostatin A
IL-1β	Interleukin-1 beta	TST	tail suspension test
IL-23	Interleukin-23	TTC	2,3,5 Triphenyltetrazolium chloride
IL-6	Interleukin-6	UC	Ulcerative colitis
iNOS	Inducible Nitric Oxide Synthase	UVB	ultraviolet radiation b
JNK	c-Jun N-terminal kinase	VEGF	Vascular endothelial growth factor
		β-MHC	beta-myosin heavy chain
